# Engineered device in *E. coli* lyses *S. aureus* at physiological fever temperatures

**DOI:** 10.17912/micropub.biology.000616

**Published:** 2022-08-01

**Authors:** Fardeen Siddiqui, Meliha Ulker, Isabelle E Laizure, Kristen C Johnson

**Affiliations:** 1 University of New Hampshire Manchester, Manchester, NH USA

## Abstract

Multiple strains of *Staphylococcus* are resistant to antibiotics, including the well-known methicillin-resistant *Staphylococcus aureus* (MRSA). We share an engineered plasmid device in *Escherichia coli* that lyses the disease-causing pathogen, *S. aureus.* The device was engineered using BioBrick parts obtained from the International Genetically Engineered Machine foundation (iGEM). The cI-blue-lysostaphin device consists of a temperature-sensitive promoter that is activated under physiological fever temperatures above 35°C that drives expression of a blue chromoprotein reporter and mature truncated lysostaphin enzyme. The functioning cI-blue-lysostaphin device was tested for optimal lysis conditions in MM294 and DH5α *E. coli* chassis and across incubation temperatures ranging from 30-42°C. We conclude that the lysostaphin activity of the cI-blue-lysostaphin device differs between chassis and increases with greater incubation temperature.

**Figure 1. Lysis of S. aureus by heat-inducible cI-blue-lysostaphin device in E. coli proceeds in a temperature-dependent manner.**
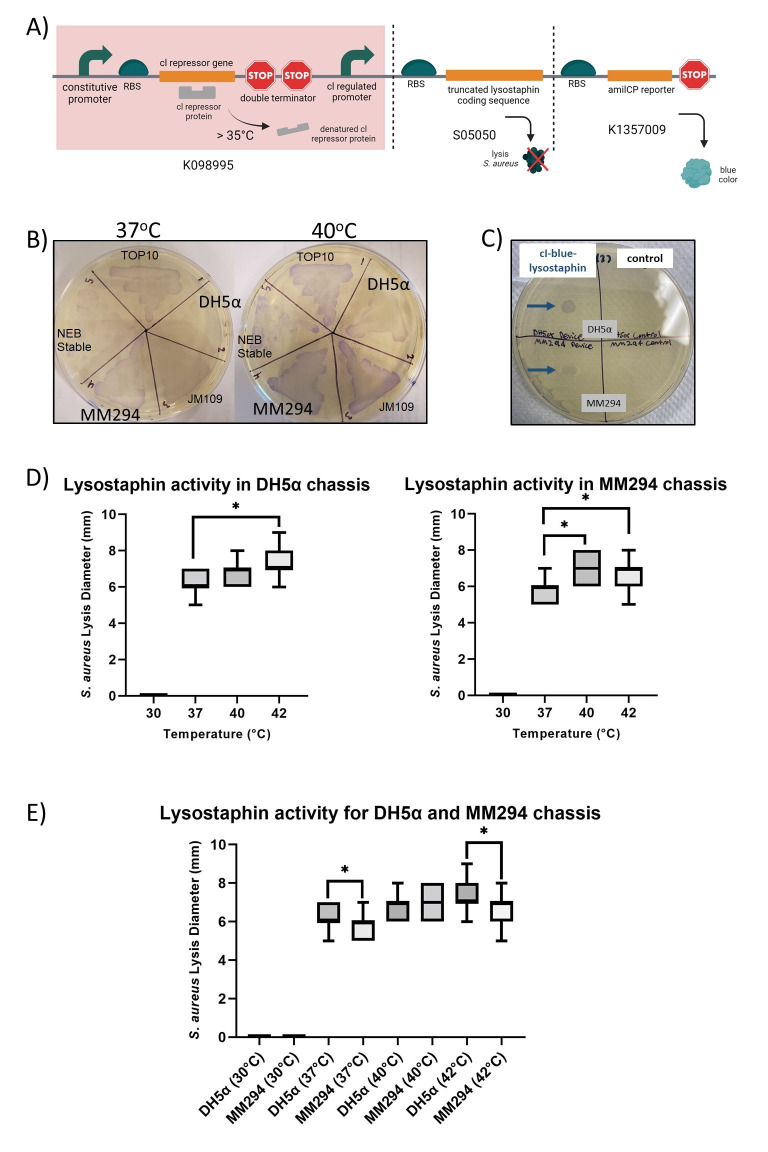
(A) cI-blue-lysostaphin device engineered from three iGEM BioBrick parts ligated together using 3A assembly. At temperatures above 35℃, the constitutively expressed cI repressor is inactivated and unable to bind to the lambda phage pR promoter thus allowing for expression of blue chromoprotein and lysostaphin in a temperature-dependent fashion. Image created in BioRender. (B) Blue chromoprotein shows highest expression in the MM294 chassis and lowest expression in the DH5α chassis at 37℃ and 40℃. cI-blue-lysostaphin was transformed into 5 different *E. coli* chassis (DH5α, JM109, MM294, NEB Stable, TOP10), inoculated into liquid cultures overnight and streaks of each culture were prepared for growth at 30℃ to 42℃ (plates shown for 37℃, 40℃). (C) Plate Lysis Assay results at 40℃ demonstrate effective lysis by cI-blue-lysostaphin containing *E. coli* (left, blue arrows) but no lysis by *E. coli* containing control (Bba_K098995 plasmid). (D) Boxplot of lysostaphin activity of the cI-blue-lysostaphin device is greater at higher temperatures in both the DH5α (left) and MM294 (right) chassis. *S. aureus* was streaked as a lawn on solid LB medium and 5μl of MM294 or DH5α culture grown overnight containing cI-blue-lysostaphin was pipetted onto the *S. aureus* lawn. Zones of inhibition (mm) were measured for all six spots of each plate following 24 hours of incubation. When all three incubation temperatures for each chassis were compared, 42℃-incubation resulted in significantly greater lysis diameter than 37℃ in the DH5α chassis (N=54, 1-way ANOVA, * *p* < 0.05). The cI-blue-lysostaphin device in MM294 also exhibited a significant increase of lysis activity at a 42℃ and a 40℃-incubation temperature compared to 37℃ (N=54, 1-way ANOVA, * *p* < 0.05). (E) Boxplot of three separate comparisons of lysis activity between the DH5α and MM294 chassis at different temperatures. At 37℃ and 42℃, cI-blue-lysostaphin had significantly better lysis activity in DH5α. N=36, Independent T-test, * *p* < 0.05.

## Description

*Staphylococcus aureus* is a ubiquitous microorganism that can be found on the surface of the skin, nose, and other areas of the body of healthy individuals (Foster 1996). *S. aureus* most commonly causes infection when the skin is punctured or broken, allowing the *S. aureus* to enter the wound. Methicillin-resistant *Staphylococcus aureus* (MRSA), *S. aureus* that has acquired resistance to β-lactam antibiotics, is responsible for high levels of morbidity and is often spread in community and healthcare settings (Klevens 2007). Increased prevalence of vancomycin-resistant MRSA strains in the last decade is a major public health concern (Wu et al. 2020). The need to find alternative treatments against *Staphylococcus* infections is an area of great interest. We propose an engineered plasmid device that produces a mature truncated lysostaphin enzyme that lyses *S. aureus* .

Lysostaphin is a monomeric zinc-containing endopeptidase that hydrolyzes bonds in the pentaglycine cross-bridge of peptidoglycan. This creates osmotically fragile *S. aureus* cells favorable for lysis conditions (Trayer & Buckley 1970, Bastos et al. 2010). The wild type lysostaphin gene has a preproenzyme that is modified extracellularly to produce mature lysostaphin. To produce mature lysostaphin, a truncated sequence can be used where the preprolysostaphin and prolysostaphin sequences are removed. (Sharma et al. 2005).

The use of lysostaphin over traditional methods of killing *S. aureus* in a clinical setting confers several advantages. Lysostaphin is a narrow spectrum antistaphylococcal enzyme that is active against both dividing and non-dividing cells while being capable of passing through the extracellular matrix to act against *S. aureus* biofilms (Jayakumar et al. 2021). Lysostaphin is also naturally digested by human intestinal proteases with no impact on gastrointestinal microbiota (Bastos et al. 2010). Lysostaphin is increasingly becoming a more suitable alternative to traditional *S. aureus* therapies and new methods are needed to advance this field of work.

Synthetic biology is the design of biological systems for beneficial purposes, including medical applications (Endy 2005). Given the gap in successful treatment of MRSA, we aimed to create a biological device that could effectively kill *S. aureus* . Utilizing BioBrick parts from iGEM, we successfully engineered a device in *E. coli* that produces mature lysostaphin when activated at physiological fever temperatures (cI-blue-lysostaphin; Figure 1A). Three BioBrick composite parts were used to create the cI-blue-lysostaphin device. The heat-sensitive cI quad part inverter (QPI) (Bba_K048995) contains a strong promoter that drives expression of a temperature-sensitive cI repressor. At temperatures above 35 ^o^ C, the cI repressor loses binding efficacy becoming unable to bind to the pR lambda phage promoter (“cI regulated promoter”) that has been placed upstream of a blue chromoprotein reporter (Bba_K1357009) and truncated (mature) lysostaphin (Bba_S05050).

To first ensure accurate assembly of the device, the sequence of the cI-blue-lysostaphin plasmid was sequence verified using Oxford Nanopore technology. The cI-blue-lysostaphin device was transformed into five different *E. coli* chassis and each was screened to determine efficacy of heat-inducible expression via visualization of the blue chromoprotein reporter (Figure 1B). MM294 and DH5α chassis were chosen for further experimentation to test their ability to lyse *S. aureus* in a heat-inducible manner.

Cultures of the MM294 and DH5α *E. coli* chassis containing the cI-blue-lysostaphin device were pipetted on *S. aureus* lawns and after incubation at temperatures ranging from 30-42°C, lysis diameter was measured. While spotting of *E. coli* containing the control plasmid (Bba_K048995) did not exhibit any lysis, effective lysis by cI-blue-lysostaphin containing *E. coli* was demonstrated at 37℃, 40℃ (Figure 1C; left, blue arrows), and 42℃. No lysis was observed at 30℃ in either chassis. Individually, cI-blue-lysostaphin in both DH5α and MM294 demonstrated a significant increase in lysis activity at a 42℃-incubation temperature compared to 37℃ (* *p* < 0.05). The cI-blue-lysostaphin device in MM294 also exhibited a significant increase of lysis activity at a 40℃-incubation temperature compared to 37℃ (* *p* < 0.05) (Figure 1D). The cI-blue-lysostaphin device in the DH5α chassis exhibited significantly greater *S. aureus* lysis than in the MM294 chassis at 37℃ and 42℃ incubation temperatures (* *p* < 0.05) (Figure 1E).

Interestingly, although initial visualization of the intensity of the blue chromoprotein reporter suggested that cI-blue-lysostaphin expression was greater in MM294 (Figure 1B), the results from the plate lysis assay indicate that the expression of lysostaphin from the cI-blue-lysostaphin device in DH5α is higher than in MM294 at most temperatures studied (Figure 1D). The explanation for these observations warrants further investigation.

cI-blue-lysostaphin did not show any lysis activity against *Staphylococcus epidermidis* when expressed in either *E. coli* chassis (extended data). This is consistent with studies that indicate the MIC _90_ of recombinant lysostaphin is 1000-10,000 times higher for *S. epidermidis* than for *S. aureus* (Wu et al. 2003).

The delivery of a treatment for *Staphylococcus* infections such as that presented here results in a few obstacles, namely the risk of *E. coli* infection. The use of the probiotic *E. coli* strain, Nissle 1917 (EcN) as the chassis for the cI-blue-lysostaphin engineered plasmid would enable a safer alternative. To prevent the acquisition of undesirable mutations and hinder the risk of infection, CRISPR-based single-chemical kill switches can be incorporated into the EcN bacterial genome (Rottinghaus et al. 2022). Presently, recombinant lysostaphin in a hydrogel or ointment can be used for superficial non implant related *Staphylococcus* infections, even those involving biofilm formation (Cheleuitte-Nieves et al. 2020). Investigation for its use in preventing implantable device-related infection is in late-stage preclinical research (Cheleuitte-Nieves et al. 2020). Intravenous injection of microsphere-encapsulated lysostaphin was demonstrated to be effective and selective in the treatment of murine MRSA-related lung infections (Xiuhui et al. 2021). It is evident that there is significant need for research to develop therapeutic agents that selectively treat MRSA without the use of antibiotics. Further research is needed to evaluate the potential of the cI-blue-lysostaphin device in the direct treatment of MRSA whether transformed into probiotic strain Nissle 1917 or adapted in another manner.

## Methods

*Plasmid Cloning*

Plasmids containing parts BBa_K098995, BBa_S05050, BBa_K1357009 and empty backbones were obtained from iGEM (http://parts.igem.org). Parts were ligated using 3A assembly (Shetty et al. 2011) to create the cI-blue-lysostaphin device shown in Figure 1A. Plasmids were transformed by Zippy Transformation of Z-competent Cells (Pope & Kent 1996) in *E. coli* MM294 and DH5α (Zymo Research, USA). Plasmid DNA was isolated with a ZymoPURE Plasmid Miniprep Kit (Zymo Research, USA). The cI-blue-lysostaphin device was sequence verified (plasmidsaurus, Eugene, OR, USA) using Nanopore sequencing on an R9.4.1 flow cell and base called using Guppy (v.6.1.5) in super-accurate (SUP) mode (Oxford Nanopore Technologies, UK). BBa_K098995 cI QPI plasmid was used as a control.

*Plate Lysis Assay*

*Staphylococcus aureus subsp. aureus* strain NCTC 8532 was streaked as a lawn on LB plates with 1.5% agar. 5μl of a saturated culture (grown overnight at 37℃) of each *E. coli* chassis (MM294 or DH5α) containing cI-blue-lysostaphin was pipetted onto the *S. aureus* lawn (n=6). Separate replicate plates were created and incubated for 24 hours at 30℃, 37℃, 40℃, and 42℃. Zones of inhibition (diameter of lysis in mm) were measured for all six spots of each plate. Control plate lysis assays with *E. coli* chassis (MM294 or DH5α) containing BBa_K09899 only were performed. Experiments were repeated on *Staphylococcus epidermidis* strain NCTC 11047 *.* Experiments were performed in triplicate. Raw data for lysis diameters are included as a supplementary excel file.

*Statistical Analysis*

A one-way between subjects ANOVA was conducted to compare the effect of temperature on cI-blue-lysostaphin lysis activity in DH5α chassis at 37℃, 40℃ and 42℃. No lysis activity was observed at 30℃, and therefore this temperature was excluded from the statistical analysis. There was a significant effect of the temperature on lysis diameter (mm) at the *p* < 0.05 level [F(2, 51) = 10.276, *p* = 0.000178]. Post hoc comparisons using the Tukey HSD test indicated that the mean lysis diameter for the 42℃ condition (M = 7.39, SD = 0.70) was significantly greater than the 37℃ condition (M = 6.39, SD = 0.61). However, there was no significant difference when comparing the difference in lysis diameter at the 40℃ (M = 6.89, SD = 0.68) to the 37℃ or 42℃ condition. These results suggest that increasing the incubation temperature led to greater lysis activity of cI-blue-lysostaphin in the DH5α chassis.

A one-way between subjects ANOVA was conducted to compare the effect of temperature on cI-blue-lysostaphin lysis activity in the MM294 chassis at 37℃, 40℃ and 42℃. No lysis activity was observed at 30℃, and therefore this temperature was excluded from the statistical analysis. There was a significant effect of the temperature on lysis diameter (mm) at the *p* < 0.05 level [F(2, 51) = 13.516, *p* = 0.000019]. Post hoc comparisons using the Tukey HSD test indicated that the mean lysis diameter for the 42℃ condition (M = 6.67, SD = 0.69) and the 40℃ condition (M = 7.00, SD = 0.84 was significantly greater than the 37℃ condition (M = 5.78, SD = 0.65). However, there was no significant difference when comparing the difference in lysis diameter at the 40℃ to the 42℃ condition. These results suggest that increasing the incubation temperature also led to greater lysis activity of cI-blue-lysostaphin in the MM294 chassis.

It should be noted that because temperature must be high to see an effect, there is a point of diminishing returns as the temperature reaches unsustainable levels for *E. coli* growth.

Three separate independent sample *T-* tests were conducted at *p* < 0.05 to compare the effect of chassis type on cI-blue-lysostaphin lysis activity (mm) at different temperatures. At 37℃, cI-blue-lysostaphin had significantly better lysis activity in DH5α (M = 6.39, SD = 0.61) compared to MM294 (M = 5.78, SD = 0.65). t(34) = 2.92, *p* = 0.003. At 40℃, cI-blue-lysostaphin did not have a significant difference in lysis activity in DH5α (M = 6.89, SD = 0.68) compared to MM294 (M = 7.00, SD = 0.84). t(34) = -0.437, *p* = 0.332. At 42℃, cI-blue-lysostaphin had significantly better lysis activity in DH5α (M = 7.39, SD = 0.70) compared to MM294 (M = 6.67, SD = 0.69). t(34) = 3.13, *p* = 0.002.

Due to cI-blue-lysostaphin becoming active at temperatures above 35℃, the experiments performed at 30℃ yielded no lysis of *S. aureus* as expected and were not included in the statistical analysis.

## Reagents

**Table:** 

**Plasmid/Strain**	**Description**	**Available From**
Bba_K098995	heat-sensitive cI quad part inverter	iGEM
Bba_S05050	Truncated mature lysostaphin	iGEM
Bba_K1357009	Blue chromoprotein	iGEM
MM294	**Genotype:** *glnX44(AS) rfbC1 endA1 spoT1 thiE1 hsdR17 creC510*	CGSC; https://cgsc.biology.yale.edu/StrainRpt.php?ID=5439
DH5α	**Genotype:** F– φ80 *lac* ZΔM15 Δ( *lac* ZYA- *arg* F)U169 *rec* A1 *end* A1 *hsd* R17(rK–, mK+) *pho* A *sup* E44 λ– *thi* -1 *gyr* A96 *rel* A1	CGSC; https://cgsc.biology.yale.edu/StrainRpt.php?ID=150015
JM109	**Genotype:** *end* A1, *rec* A1, *gyr* A96, *thi, hsd* R17 (r _k_^–^ , m _k_^+^ ), *rel* A1, *sup* E44, Δ( *lac-pro* AB), [F´ *tra* D36, *pro* AB, *lac* I ^q^ ZΔM15]	Promega
TOP10	**Genotype:** F– *mcr* A Δ( *mrr* - *hsd* RMS- *mcr* BC) φ80 *lac* ZΔM15 Δ *lac* X74 *rec* A1 *ara* D139 Δ( *ara-leu* )7697 *gal* U *gal* K λ– *rps* L(StrR) *end* A1 *nup* G	ThermoFisher
NEB Stable	**Genotype:** F' *proA+B+ lacI ^q^ ∆(lacZ)M15 zzf::Tn10* (Tet ^R^ )/ *∆(ara-leu) 7697 araD139 fhuA ∆lacX74 galK16 galE15 e14- Φ80dlacZ∆M15 recA1 relA1 endA1 nupG rpsL* (Str ^R^ ) *rph spoT1 ∆(mrr-hsdRMS-mcrBC)*	New England Biolabs
*Staphylococcus epidermidis*	strain NCTC 11047	ATCC
*Staphylococcus aureus subsp. aureus*	strain NCTC 8532	ATCC

## Extended Data

Description: Plate Lysis Assay Raw Data. Resource Type: Dataset. DOI: 10.22002/D1.20244
